# Low-temperature threshold for egg survival of a post-diapause and non-diapause European aedine strain, *Aedes albopictus* (Diptera: Culicidae)

**DOI:** 10.1186/1756-3305-5-100

**Published:** 2012-05-23

**Authors:** Stephanie Margarete Thomas, Ulla Obermayr, Dominik Fischer, Juergen Kreyling, Carl Beierkuhnlein

**Affiliations:** 1 Department of Biogeography, University of Bayreuth, Universitaetsstrasse 30, D-95447, Bayreuth, Germany; 2 Biogents AG, Department of Zoology, University of Regensburg, Universitaetsstrasse 31, D-93053, Regensburg, Germany

## Abstract

**Background:**

The interplay between global warming and invasive arthropods in temperate zones is of utmost interest in terms of the potential expansions of vector-borne diseases. Up to now, investigations on the recent establishment of mosquito vectors have focused on temperatures during their phases of activity. However, cold temperatures may also act as a strong ecological constraint. Projected changes in winter climate indicate an increase of mean minimum temperatures of the coldest quarter, less frequent days with frost and a shorter frost-season in Europe at the end of the century. Nevertheless, single cold extremes are also expected to persist under warming scenarios, which have a strong impact on reproduction success.

**Methods:**

Here, the temperature constraints of European *Aedes albopictus* eggs, which had passed through a diapause, compared to non-diapausing eggs were examined systematically under controlled laboratory conditions. Additionally, one tropical strain of *Ae. albopictus* and of *Ae. aegypti* was used in the comparison.

**Results:**

The lower temperature threshold tolerated by the European eggs of *Ae. albopictus* which have undergone a diapause, was -10°C for long term exposures (12 and 24h) and -12°C for 1h exposure. Non-diapausing eggs of European *Ae. albopictus* were found to hatch after a -7°C cold treatment (8, 12 and 24h exposure). Both tropical aedine species only tolerated the long term treatment at -2°C. Neither *Ae. albopictus* nor *Ae. aegypti* eggs hatched after being exposed to -15°C. Survival was mainly influenced by temperature (F = 329.2, df = 1, p < 0.001), whereas the duration of the cold treatment only significantly influenced the hatching response at the thermal limits of survival (F = 5.6, df = 1, p = 0.031) but not at 0°C (F = 0.1, df = 1, p = 0.730). Hatching success after the cold treatment was significantly increased in European eggs, which have undergone a diapause compared to non-diapausing eggs (F = 14.7, df = 3, p < 0.001). These results illustrate rapid adaptation.

**Conclusions:**

Here, low temperature thresholds for aedine mosquito egg survival were detected. The compilation of risk maps for temperate regions can substantially be improved by considering areas where an establishment of a vector population is unlikely due to winter conditions.

## Background

The interplay between invasive arthropods and global warming is of utmost interest in terms of potential expansions or shifts of vector-borne infectious diseases [1,2]. In Europe, six non-European aedine mosquito species have been found, quite recently *Aedes koreicus*[3,4]. Almost all were accidentally imported by the used tire trade [4]. *Aedes albopictus* (Diptera: Culicidae) is a prominent example of an invasive arthropod vector. It is a competent vector for 22 arboviruses (among others Chikungunya and dengue) and for *Dirofilaria* worms [5]. Almost 30 years after the first recorded introduction of this vector to Europe, the first autochthonous cases of Chikungunya [6] and dengue [7] have been detected in southern Europe. Originating from subtropical and tropical regions, the aedine vector has overcome a wide range of continental and oceanic barriers during the last decades [8]. Today, among other regions [9] the European continent is affected by the invasion of this species. Laboratory experiments [10-12] and field observations [13,14] on the climatic constraints and on the climate-driven population dynamics of this mosquito have focused on the role of temperature during the phase of larval and adult activity, but rarely during the total life cycle [15]. Mortality of eggs has been examined under different levels of relative humidity and warm temperatures [16]. However, low temperatures during inactive periods may also act as an ecological constraint for the range expansion of mosquitoes [17]. In the case of shifts of populations to higher latitudes, the minimum temperatures and the mean temperature of the coldest month or quarter have been discussed as a proxy for the ecological constraints of *Ae. albopictus*[18]. But sensitivity against temperature thresholds and the ability to cope with thermal constraints is not a permanent trait during the life cycle of this species. The capability to develop cold-tolerant diapausing eggs under specific environmental conditions is one key factor for *Ae. albopictus* to establish in higher latitudes [19]. Photoperiod and temperature are known to modulate the production of diapausing and non-diapausing eggs in *Ae. albopictus*[13,20]. During diapause, desiccation resistance in eggs increases due to higher concentrations of hydrocarbons at the egg surface [21].

Projected changes in winter climate indicate an increase of mean minimum temperatures of the coldest quarter by 2.2-4.2°C in south-western Europe, and by up to 4.0-6.4°C in northern Europe (IPCC scenario A2, 1961–1990 to 2071–2100) [22]. However, these values do not display changes in absolute minimum temperatures. Furthermore, less frequent days with frost (a decrease of 60-80% and 30-45%, in south-western and northern Europe, respectively) and a shorter frost-season (a decrease of 35-50% and 20-30%, in south-western and northern Europe, respectively) are expected at the end of the century [22]. Nevertheless, single cold extremes seem to persist by the end of this century also under warming scenarios because of increasing climatic variability [23,24]. These cold spells are usually short term events with a strong impact on the population dynamics of mosquitoes and thus on mosquito-borne disease transmission. Effects are expected especially when late frost events happen to occur in spring [25]. Hence, the influence of short frost events on egg survival when mosquito diapause is already broken is highly relevant for populations to establish and in terms of population dynamics.

Up to now, investigations on the recent establishment of the mosquito vector focused on temperatures during the phases of activity [10-14,16]. However, cold temperatures may also act as a strong ecological constraint in terms of possible range expansions [18]. Winter warming may increase the survival rate of mosquito eggs, but as evidence suggests that climatic variability and extreme temperature events will become more important, reproduction success and population dynamics can also be negatively affected [26]. As a matter of fact, the minimum temperature constraints of European *Ae. albopictus* eggs have not yet been systematically examined under controlled laboratory conditions. Because regional invasive populations of the species are known to adapt rapidly to the climatic conditions such knowledge on low temperature constraints is urgently needed. Here, we hypothesized that: (1) the eggs of European *Ae. albopictus* which have undergone a diapause tolerate minimum temperatures down to -10°C, (2) the duration of exposure to frost influences the survival of eggs and (3) hatching success after a cold treatment increases in eggs which have passed through a diapause compared to non-diapausing eggs.

## Methods

### Species, strains and standard protocol

Overall, we tested eggs from two strains of *Ae. albopictus* (temperate European and tropical Asian origins) and one strain of *Ae. aegypti* (tropical Asian origin). The European *Ae. albopictus* strain originated from eggs collected in the field in Rimini (Italy) and reared in the laboratory (Rimini F43). The tropical aedine species were obtained from a long-lasting laboratory colony. Laboratory colonies were used so that further threshold experiments with the same strains could be combined as a basis for epidemiological and environmental modeling.

The temperate strain of *Ae. albopictus* was artificially introduced to diapause. For all strains, we determined the survival of eggs after being exposed to low temperatures. In a climate chamber trial we applied a gradient of minimum temperatures (0 to -15°C). Each temperature treatment was replicated for different durations (1 to 24h).

Female mosquitoes were reared according to the standard protocol at a temperature of 27°C, a relative humidity of 60 – 80% and a 12:12 h (L:D) photoperiod. The light period was set from 8:00 to 20:00 and held constant at 150 Lux. After hatching from the eggs, larvae were kept in a water basin (30 x 30 x 10cm) that was filled with a 1:1 mixture of tap- and deionised water. Larvae were fed with fishfood flakes (Tetra Min®). Before adult emergence, the pupae were transferred to a cage (40 x 30 x 20cm). Adult mosquitoes had constant access to sugar solution (10% dextrose). At the age of seven to ten days after emergence from the pupae, females received their first blood-meal with human blood derived from expired blood preservations. The blood was heated up to 38 ± 1°C and transferred into sheep intestines to be exposed to the mosquito cage population. After three to four days eggs were laid on moistened filter paper, subsequently removed from the cage, left to dry for 2 days and then stored in plastic bags at 27°C with a minimum relative humidity of 85% before being exposed to the frost manipulation.

### Induction of diapausing eggs

In *Ae. albopictus*, the production of diapausing eggs is known to be induced by low temperatures and shortened photoperiods [27,28]. According to Hanson and Craig [29], the induction of diapause under laboratory conditions can be achieved by transferring mosquito pupae from rearing conditions at 27°C and a 16:8 h (L:D) photoperiod to 21°C and a 8:16 h (L:D) photoperiod. We applied this procedure with some minor modifications: After hatching from the eggs, European *Ae. albopictus* larvae were kept at a temperature of 27°C, a relative humidity of 60 – 80% and a 12:12 h (L:D) photoperiod until they reached the second instar. The basins were then transferred to a room at 21-22°C, 30-40% rH and a 8:16 h (L:D) photoperiod [29]. The light period (150 Lux) was set from 8:00 to 16:00. In contrast to [29], mosquito larvae were kept at 21-22°C and at a 8:16 h (L:D) photoperiod as soon as they reached the second instar larval stage. By doing so the chances of diapause induction are increased while overall development is slowed down. Before emergence the pupae were transferred to a cage (40 x 30 x 20 cm) and provided with sugar solution (10% dextrose). Adults were held under the same conditions and were fed blood after 7-12 days. Eggs were laid on moistened filter paper after three to four days and left to dry for 2 days. They were then stored for 14 weeks in plastic bags at 21-22°C with a minimum relative humidity of 60% until spontaneous hatching occurred again with about 90%.

### Treatment

We manually placed 20 eggs (with each egg inspected under a stereo microscope to exclude capped eggs or those with signs of desiccation) from each *Aedes* population onto a moist filter paper pad, which was then deposited in a glass vial. This vial was then sealed by a rubber plug to reduce the loss of moisture and, hence to minimize any possible desiccation effects. The experimental design aimed to maximize temperature levels and cold duration in order to determine lethal thresholds as precisely as possible. The eggs were exposed to seven different temperatures: 0°C, -2°C, -5°C, -7°C, -10°C, -12°C and -15°C by using a climate chamber (Licht-Thermostate Typ 1301, RUMED, Rubarth Apparate, Laatzen, Germany). The duration was 1, 4, 8, 12 and 24h (16:8 (L:D) photoperiod) for each temperature treatment, respectively.

### Hatching and counting

After the cold treatment, the eggs were acclimated at room temperature (18-20°C). The glass vials containing the filter paper pad with 20 eggs were filled with 20 ml of nutrient broth solution (cooled boiled water with the addition of powdered bacto-nutrient broth, 0.1% in weight, according to Novak and Shroyer [30]). Furthermore, the temperature of the nutrient broth solution was set to 25°C to stimulate the hatching response. The submerged eggs of the tropical strains were kept at 25°C with a 16:8 h (L:D) photoperiod and the eggs of the temperate strains were kept at 20°C. Hatching at each level of temperature was determined after 12, 24, 48, 72 and 96h. Larvae were removed at these times, respectively. The minimum survival temperature was quantified as the lowest temperature at which successful hatching was observed. The hatching rate was also quantified as a percent for each combination of temperature and duration.

### Statistics

The interaction between temperature level and the duration of exposure on survival was explored by a linear least squares regression of the minimum survival temperature for each category of duration. Inter-specific differences in minimum survival temperature were compared by analyzing the slopes of the linear regression between the duration and the minimum survival temperature. This was evaluated by a Monte-Carlo permutation procedure to compare the true difference in the slope of two strains with the differences in 1,000 permutations of randomly-assembled groups. The importance of temperature and duration on hatching success was further investigated by linear models while using the species/strain identity as a co-variate. Differences between species/strains were evaluated by the same linear models combined with analyses of variance (anova).

Prior to statistical analysis, data were log-transformed if conditions of normality were not met or to improve the homogeneity of variances. Both characteristics were tested by examining the residuals versus fitted plots and the normal qq-plots of the linear models. Statistical analysis was carried out using R 2.12.0 [31] supported by the package simba [32].

## Results

### Minimum survival temperature

**Figure 1:**
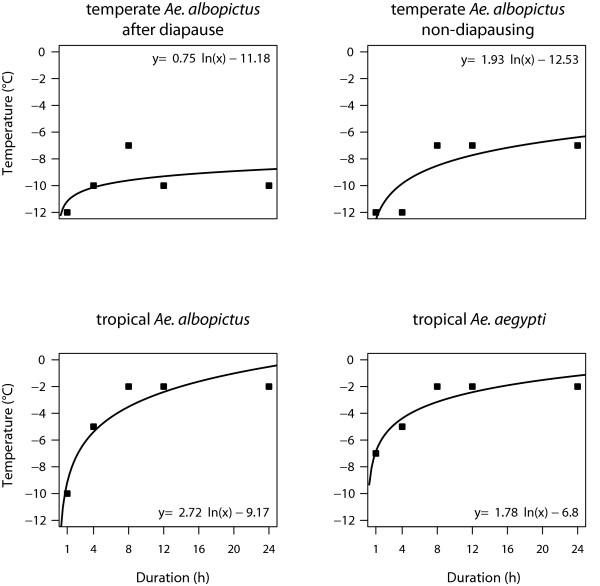
**Minimum survival temperature of*Aedes*eggs after cold treatment.** Minimum survival temperature in comparison to duration of treatment for European eggs which have undergone a diapause and non-diapausing eggs of *Ae. albopictus* and tropical eggs of *Ae. albopictus* and *Ae. aegypti*. Eggs of European *Ae. albopictus* which passed through a diapause survive lower minimum temperatures than non-diapausing eggs when exposed for more than 12h, whereas no differences occurred for short exposure (1h). Eggs of tropical *Ae. albopictus* survive lower minimum temperatures than *Ae. aegypti* eggs when exposed for 1h, whereas no differences occurred for longer exposure (>8h).

The minimum survival temperature tolerated by the European eggs of *Ae. albopictus* after a diapause (Figure 1) was -10°C for long term exposures (12 and 24h), while they survived short term exposure (1h) at -12°C. Non-diapausing eggs of European *Ae. albopictus* were found to hatch even after a 4h treatment at -12°C. However, with mean and long term exposures (8, 12 and 24h) hatching only occurred at less extreme temperatures (-7°C). The tropical aedine species only differed in its hatch response for the 1h cold treatment: While tropical *Ae. albopictus* survived at -10°C, *Ae. aegypti* hatched at -7°C. In contrast to the short term exposure to cold temperatures, both species only tolerated the long term treatment at -2°C. The minimum survival temperature for different durations of exposure did not differ significantly between strains/species according to the Monte-Carlo permutation procedure of the slopes of the linear regression.

### Duration of exposure

Survival was mainly influenced by temperature (F = 329.2, df = 1, p < 0.001). In comparison to temperature, the duration of exposure only had a minor impact on the hatching rate (F = 16.2, df = 1, p < 0.001). The duration of the cold treatment only significantly influenced the hatching response at the thermal limits of survival for each strain/species (F = 5.6, df =1, p = 0.031) but not at 0°C (F = 0.1, df =1, p = 0.730).

### Hatching success after a cold treatment

**Figure 2:**
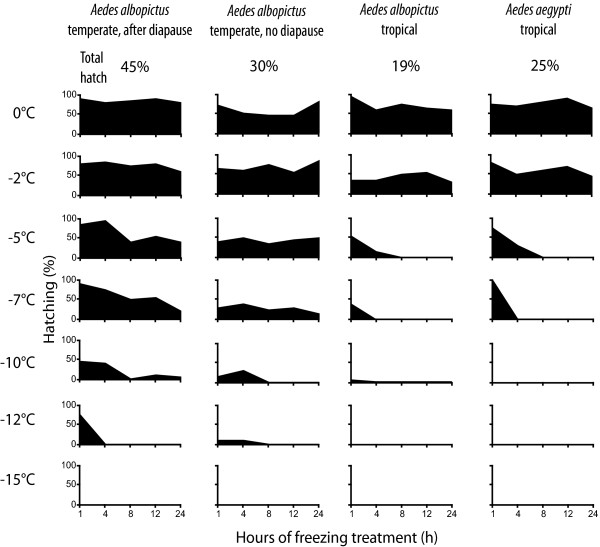
**Hatching success of Aedes eggs after cold treatment.** Hatching success of *Aedes* after exposure to cold temperatures (0°C to -15°C) for different durations (1 to 24h).

Hatching success after the cold treatment was significantly increased in European eggs that have undergone a diapause compared to non-diapausing eggs (F = 14.7, df = 3, p < 0.001; Figure 2). Differences within the same geographical range but between species (tropical *Ae. albopictus* and *Ae. aegypti*) were less pronounced than differences between geographically different strains of the same species (tropical and European *Ae. albopictus*). Hatching success of European *Ae. albopictus* after the total cold treatment was 45% for eggs after diapause and 30% in non-diapausing eggs. Only eggs of the European strain hatched after exposure to -12°C for 1h: Eggs after a diapause showed a high‐hatching success (75%), where as hatching success was decreased in non-diapausing eggs (10%). Tropical *Ae. albopictus* and *Ae. aegypti* total hatching success was 19 and 25%, respectively. Surprisingly, hatching of one tropical *Ae. albopictus* egg occurred after exposure to -10°C for 1h. The hatching success of *Ae. aegypti* was almost unaffected when exposed to -7°C for 1h, whereas an extension of the -7°C cold period for more than 1h or a further reduction of temperature down to -10°C caused a complete breakdown of hatching. Neither *Ae. albopictus* nor *Ae. aegypti* eggs hatched after being exposed to -15°C.

## Discussion

We determined the minimum survival temperature of eggs for two strains of *Ae. albopictus* and for one strain of *Ae. aegypti* and showed that the hatching success after the cold treatment was significantly increased in European eggs which have undergone a diapause compared to non-diapausing European eggs after exposure to cold temperatures (0°C to -15°C), for different durations. Overall, we focused on relatively short exposure times (1h, 4h, 8h, 12h and 24h), because these are the time spans on which minimum temperatures are available from climatology and meteorology. Consequently, our data can be used to make evidence-based decisions on the temporal resolution of temperature data needed for modeling approaches. Temperature was the main controlling factor, whereas the duration of the cold treatment only influenced the hatching response significantly at the thermal limits of survival for each strain/species.

To the best of our knowledge there are no comparable studies on the cold tolerance in *Ae. albopictus* eggs which have undergone a diapause. Previous studies on non-diapausing, non cold-acclimated eggs of a North American strain of *Ae. albopictus* have shown that no hatching occurs after a 24h cold period of -10°C [18]. Our results confirmed this threshold for at which no eggs survive European non-diapausing *Ae. albopictus* eggs. However, the survival of the European strain in this study is reduced more strongly with decreasing temperature than the survival of the North American: for the European strain we found 50% egg mortality at -5°C, whereas for the North American strain 50% egg mortality at -8°C was observed [33]. The minimum survival temperature of *Ae. aegypti* eggs in the field (Houston, Texas, USA) was found to be 7°C for a 24h cold period [33]. But in nature short term temperature fluctuations occur. Additionally, at one sampling site small scale differences across short distances can be substantial. Ecological complexity contributes to statistical noise. This is why controlled experiments are needed to identify physiologically relevant thresholds.

In the laboratory -3°C for 24h was identified as a threshold for *Ae. aegypti* eggs, respectively [33]. The present results show only a slight increase in the minimum survival temperature for *Ae. aegypti* (-2°C for 24h). Using a very long-lasting laboratory colony may yield deviating results compared to natural populations. However, such colonies are more appropriate to serve for experimental proof of life cycle thresholds.

It was suggested that the duration of exposure to temperatures below a certain value is an important factor influencing the hatching rate of mosquito eggs [18]. Cold acclimation tends to increase cold hardiness in *Ae. albopictus* eggs [29,33]. In this study, previous cold acclimation was avoided to distinguish between the direct impact of cold temperatures and the duration of exposure (see also [34]). The role of the duration of low temperatures is confirmed at the thermal limits of egg survival by the present study.

Up to now, the risk of *Ae. albopictus* to establish in Europe was assumed to be relevant only for regions with cold-month mean temperature of 0°C or higher [35,36]. The -5°C coldest-month isotherm was suggested to characterize the maximum northward expansion for continental Asia and also for North America [37]. The results presented in this study emphasize the ecological importance of absolute minimum temperatures. Up to now, thermal minima are neither adequately considered in environmental niche models nor in epidemiological models. Yet, vector niche modeling is mainly based on long-term average conditions such as annual mean temperature and annual precipitation (e.g. [38]). Winter conditions in terms of the mean minimum temperature of the coldest month and days with ground frost per month are considered in the niche model of *Ae. albopictus*[39]. Although the incorporation of the absolute minimum temperatures would considerably improve vector risk maps, this would be difficult to implement in large scale projections due to the limited availability of meteorological data in an hourly resolution. Recent epidemiological models consider temperature and season dependent population dynamics of vectors [40]. However, those models start each annual cycle with the same initial number of mosquito individuals. Knowing the minimum survival temperature and survival success after frost events has therefore the potential to improve epidemiological models in temperate zones substantially.

The present study has certain specifics that need to be taken into account: First, the long colonization history of the European *Ae. albopictus* strain raises the question to what extent this strain actually now represents the characteristics of its founding population. Second, the successful production of diapausing eggs in the laboratory in all females under the recorded circumstances is unlikely. Hatching tests during diapause still showed about 10% spontaneous hatching.

Nonetheless, winter conditions, or more specifically absolute minimum temperatures, play a decisive role for the distribution limits of a species [41]. Spatial quantification of absolute minimum temperatures, however, is non-trivial. Microclimate may vary up to 10°C at small spatial scales [42]. The specific thermal conditions of microsites were found to matter also for the occurrence of aedine species [43]. For these species, indoor breeding sites [44] are adding anthropogenic habitats with specific conditions that do not refer to landscape traits. Thus, it would be naive to concentrate on natural and semi-natural site conditions alone.

Future studies should pay attention to winter conditions by incorporating realistic freeze-thaw cycles to identify how far these temperature fluctuations are affecting egg survival. Furthermore, the knowledge on winter survival of viruses such as dengue and Chikungunya within the eggs of vector insects is of utmost importance and should be intensively addressed in future research.

## Conclusions

Until now, low temperatures have not been considered adequately in the modeling of vector species. Here, low temperature thresholds for aedine mosquito egg survival were detected. The compilation of risk maps for temperate regions can substantially be improved by considering areas where an establishment of a vector population is unlikely due to winter conditions (see Additional file 1).

## Competing interests

The authors declare that they have no competing interests.

## Authors’ contributions

ST participated in the design of the study, carried out the cold treatment of eggs, performed the statistical analysis and drafted the manuscript. UO carried out the induction of diapause in mosquito eggs. DF helped to draft the manuscript. JK conceived of the study and participated in the performance of the statistical analysis. CB coordinated the study and helped to draft the manuscript. All authors read and approved the final manuscript.

## Supplementary Material

Additional file 1: Surface air temperature at night in Europe on one of the coldest nights in winter 2011, which was one of the coldest winters of the last decade (2011-02-23; http://daac.gsfc.nasa.gov/giovanni, [45]). This map shows where the distribution limits of aedine species in Europe are to be expected due to their minimum survival temperature of eggs. In the experiment, European diapausing *Aedes albopictus* survived -10°C, European non-diapausing *Ae. albopictus* -7°C, tropical *Ae. albopictus* and *Aedes aegypti* -2°C for 12h. Obviously, there is only a narrow margin between regions with a possible winter survival of non-diapausing eggs and those where winter survival of diapausing eggs seems to be possible. Click here for file
