# Supplementary figures and images for: Low-temperature threshold for egg survival of a post-diapause and non-diapause European aedine strain, Aedes albopictus (Diptera: Culicidae)
Source: Parasit Vectors. 2012 May 23;5:100. doi: 10.1186/1756-3305-5-100 (PMC3403971; doi:10.1186/1756-3305-5-100)

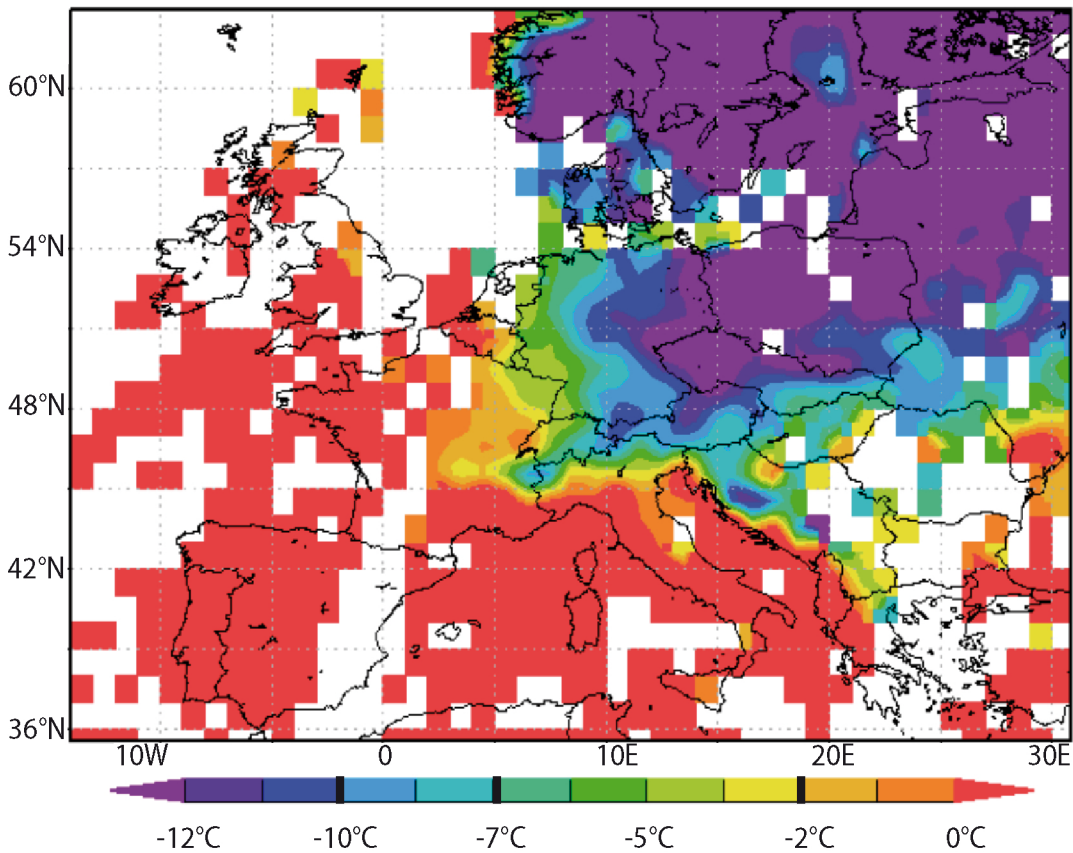

Supplement: Additional file 1: — Surface air temperature at night in Europe on one of the coldest nights in winter 2011, which was one of the coldest winters of the last decade (2011-02-23; http://daac.gsfc.nasa.gov/giovanni, [45]). This map shows where the distribution limits of aedine species in Europe are to be expected due to their minimum survival temperature of eggs. In the experiment, European diapausing Aedes albopictus survived -10°C, European non-diapausing Ae. albopictus -7°C, tropical Ae. albopictus and Aedes aegypti -2°C for 12h. Obviously, there is only a narrow margin between regions with a possible winter survival of non-diapausing eggs and those where winter survival of diapausing eggs seems to be possible. [file 1756-3305-5-100-S1.pdf]
